# Investigation of the Role of Protein Kinase D in Human Rhinovirus Replication

**DOI:** 10.1128/JVI.00217-17

**Published:** 2017-04-13

**Authors:** Anabel Guedán, Dawid Swieboda, Mark Charles, Marie Toussaint, Sebastian L. Johnston, Amin Asfor, Anusha Panjwani, Tobias J. Tuthill, Henry Danahay, Tony Raynham, Aurelie Mousnier, Roberto Solari

**Affiliations:** a National Heart and Lung Institute, Imperial College of Science, Technology and Medicine, London, United Kingdom; b The Pirbright Institute, Pirbright, Surrey, United Kingdom; c CRT Discovery Laboratories, Cambridge, United Kingdom; d Enterprise Therapeutics, Sussex Innovation Centre, University of Sussex, Brighton, United Kingdom; e Centre for Experimental Medicine, Queen's University Belfast, Belfast, United Kingdom; University of Texas Southwestern Medical Center

**Keywords:** Golgi membrane, protein kinase D, antiviral, picornavirus, rhinovirus, viral replication

## Abstract

Picornavirus replication is known to cause extensive remodeling of Golgi and endoplasmic reticulum membranes, and a number of the host proteins involved in the viral replication complex have been identified, including oxysterol binding protein (OSBP) and phosphatidylinositol 4-kinase III beta (PI4KB). Since both OSBP and PI4KB are substrates for protein kinase D (PKD) and PKD is known to be involved in the control of Golgi membrane vesicular and lipid transport, we hypothesized that PKD played a role in viral replication. We present multiple lines of evidence in support of this hypothesis. First, infection of HeLa cells with human rhinovirus (HRV) induced the phosphorylation of PKD. Second, PKD inhibitors reduced HRV genome replication, protein expression, and titers in a concentration-dependent fashion and also blocked the replication of poliovirus (PV) and foot-and-mouth disease virus (FMDV) in a variety of cells. Third, HRV replication was significantly reduced in HeLa cells overexpressing wild-type and mutant forms of PKD1. Fourth, HRV genome replication was reduced in HAP1 cells in which the PKD1 gene was knocked out by clustered regularly interspaced short palindromic repeats (CRISPR)-Cas9. Although we have not identified the molecular mechanism through which PKD regulates viral replication, our data suggest that this is not due to enhanced interferon signaling or an inhibition of clathrin-mediated endocytosis, and PKD inhibitors do not need to be present during viral uptake. Our data show for the first time that targeting PKD with small molecules can inhibit the replication of HRV, PV, and FMDV, and therefore, PKD may represent a novel antiviral target for drug discovery.

**IMPORTANCE** Picornaviruses remain an important family of human and animal pathogens for which we have a very limited arsenal of antiviral agents. HRV is the causative agent of the common cold, which in itself is a relatively trivial infection; however, in asthma and chronic obstructive pulmonary disease (COPD) patients, this virus is a major cause of exacerbations resulting in an increased use of medication, worsening symptoms, and, frequently, hospital admission. Thus, HRV represents a substantial health care and economic burden for which there are no approved therapies. We sought to identify a novel host target as a potential anti-HRV therapy. HRV infection induces the phosphorylation of PKD, and inhibitors of this kinase effectively block HRV replication at an early stage of the viral life cycle. Moreover, PKD inhibitors also block PV and FMDV replication. This is the first description that PKD may represent a target for antiviral drug discovery.

## INTRODUCTION

Picornaviruses are a family of nonenveloped, single-positive-strand RNA viruses with a genome of about 7.5 kb. This family includes a number of important human and animal pathogens, including poliovirus (PV), foot-and-mouth disease virus (FMDV), hepatitis A virus, coxsackievirus, and human rhinovirus (HRV). HRV causes the common cold in humans, which is a relatively trivial infection in healthy individuals; however, there is a clear link between HRV infection, the development of asthma and allergies, and acute exacerbations of symptoms in asthma and chronic obstructive pulmonary disease (COPD) patients (1–3). Consequently, HRV infection represents a major health care problem in a large group of individuals, and in terms of economic burden, systematic reviews reveal that between 47 and 86% of direct asthma costs can be attributed to inpatient hospitalizations (4). There are currently thought to be about 160 HRV strains, and there is no vaccine or antiviral therapeutic. For this whole family of viruses, there are currently vaccines for PV, hepatitis A virus, FMDV, and enterovirus 71 (EV71), but after decades of research, there are still no approved antiviral drugs despite the significant unmet need.

Picornavirus replication occurs in the cytoplasm. Following cell entry, the HRV genome is translated into a polyprotein that is posttranslationally processed by virus-encoded proteases into structural (capsid) proteins and nonstructural proteins, the latter including several functional intermediates and 7 mature proteins that are all required for viral replication. A number of these viral proteins have been the subject of historical drug discovery efforts, including capsid, 3C protease, and RNA-dependent RNA polymerase (5–7). These viral targets have the potential advantage of avoiding cellular toxicity but face the challenge of the emergence of virus resistance due to mutation. Consequently, attention has recently turned to the alternative strategy of identifying host targets required for viral replication.

It is well documented that picornaviruses use the cytoplasmic face of the endoplasmic reticulum (ER) and Golgi membranes for genome replication, and the morphology of these membranes is greatly remodeled by the replicating virus (8, 9). Studies on the picornavirus nonstructural proteins 2B, 2C, and 3A have shown that they associate with the ER and Golgi membranes, and it has been suggested that these proteins play an important role in membrane protein and lipid remodeling to generate viral replication complexes. A number of host proteins have been identified as being involved in the enteroviral replication complex, including phosphatidylinositol 4-kinase III beta (PI4KB) (10–18), oxysterol binding protein (OSBP) (19–22), and the Golgi membrane-specific brefeldin A resistance guanine nucleotide exchange factor 1 (GBF1) of ADP ribosylation factor 1 (Arf1) (12, 23–27). Inhibitors of these targets have been shown to effectively inhibit picornavirus replication (10–27), although there are notable differences between viruses, such as FMDV, which does not appear to depend on PI4KB (28, 29). Other potential components of the replication complex have been identified by proteomics analyses (30), including vesicle-associated membrane protein (VAMP)-associated protein A (VAP-A), an ER protein which binds and regulates OSBP (31, 32). The interaction of VAP-A with OSBP is blocked by the antiviral effector interferon (IFN)-induced transmembrane protein 3 (IFITM3), which disrupts cholesterol trafficking and viral entry (33). In uninfected cells, protein kinase D (PKD) is recruited to the Golgi membrane by Arf1 and diacylglycerol (DAG) (34, 35) and is an upstream regulator of PI4KB, OSBP, and ceramide transfer protein (CERT) (36–40). Ceramide transport to the Golgi membrane provides the substrate for sphingomyelin synthase (SGMS), which generates DAG and recruits PKD (41). At the *trans*-Golgi network (TGN), PKD activates PI4KB to generate phosphatidylinositol 4-phosphate (PI4P), which mediates the Golgi membrane localization of CERT and OSBP through their pleckstrin homology (PH) domains, and PKD phosphorylation of CERT and OSBP inhibits their functions (36–38). PKD is also present along with Arf1, PI4KB, 14-3-3γ, and C-terminal binding protein/brefeldin A-ADP-ribosylated substrate (CtBP/BARS) in a complex that mediates vesicle budding and fission from the Golgi membrane (42). Thus, PKD may be critical for regulating Golgi membrane remodeling and fission events and the control of lipid homeostasis (40), events considered critical for the formation of viral replication complexes (43).

PKD is related to the protein kinase C (PKC) family (it was originally called PKCμ) and can be directly activated by DAG, but it is also downstream and activated by PKCε. There are three members of the PKD family, and they all have an N-terminal regulatory domain with a tandem repeat of zinc finger-like cysteine-rich domains (CRDs), which bind DAG. These domains are followed by a PH domain, which binds cellular lipids, followed by a Ser/Thr kinase domain. Although first classified as a novel member of the PKC family, the kinase domain most closely resembles the myosin light chain kinase and Ca^2+^/calmodulin-dependent protein kinase (CAMK); hence, it has been reclassified as a member of the CAMK family (44). Studies have shown that PKD is activated by PKCε at S744 and S748 in the kinase activation loop, resulting in kinase activation and autophosphorylation of PKD at S916 located at the C terminus (45).

PKD can be activated by multiple upstream pathways. First, PKC can be activated by multiple receptors that generate DAG through phospholipase C (PLC) and activate the PKC pathway. Second, PKD can be activated during apoptosis through cleavage by caspase (46). Third, PKD in the Golgi membrane can be activated by Gβγ subunits (47). PKD has been implicated in several biological functions, which include the proliferation, apoptosis, and invasion of cancer cells, which has made PKD an attractive target for cancer drug discovery (47–51).

Although there are no reports of an involvement of PKD in picornavirus replication, previous studies suggested that PKD is activated by the type I interferon receptor (IFNAR), which is then phosphorylated by PKD2 in a feedback loop leading to IFNAR internalization and downregulation. This PKD2-mediated negative-feedback loop was proposed to suppress IFNAR signaling, and therefore, it was suggested that PKD inhibitors may be antiviral by boosting IFN signaling (52, 53). We therefore sought to explore these hypotheses and demonstrate that the manipulation of PKD activity by three different techniques can have a profound effect on HRV replication, acting at an early stage in the replication cycle but independently of the IFN signaling mechanism.

## RESULTS

### PKD activation following HRV infection.

**FIG 1:**
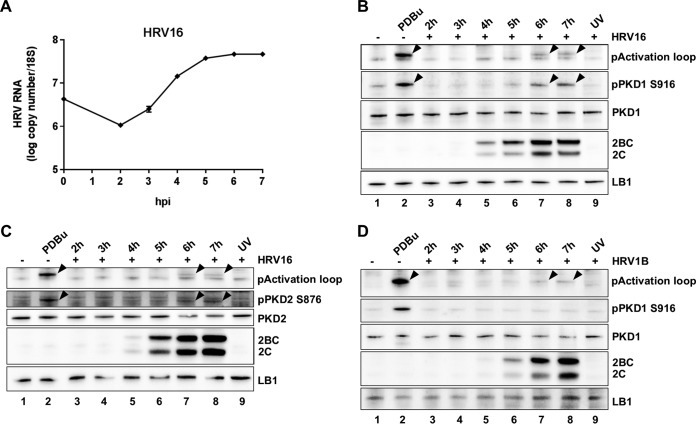
PKD phosphorylation during HRV infection. (A) The viral RNA level was measured by qRT-PCR and normalized to the 18S RNA level during the time course of infection. The graph shows the means (±SEM) of data from three independent experiments. (B) HeLa cells were infected with HRV16 at an MOI of 20 for 1 h, followed by a 7-h replication time course. Cell extracts were prepared every hour at 2 to 7 hpi (lanes 3 to 8) and analyzed by Western blotting. Uninfected cells are shown in lane 1, and uninfected cells treated with PDBu are shown in lane 2. Cells infected for 7 h with UV-inactivated virus are shown in lane 9. The blots from each experiment were immunostained with antibodies to phospho-PKD1/2 S744/S748 (pActivation loop), phospho-PKD1 S916 (pPKD1 S916), PKD1, HRV 2C, and lamin B1 (LB1). Arrowheads reveal the specific phosphorylated band. (C) A similar analysis was performed in an independent experiment with HeLa cells infected with HRV16 and immunostained with an antibody specific to phospho-PKD2 S876 (pPKD2 S876) and PKD2. (D) A similar experiment was performed in HBECs infected with HRV1B at an MOI of 20. Results of all experiments are representative of data from three independent repeats.

In order to test the hypothesis that PKD may be involved in HRV replication, we first infected HeLa cells with HRV16 and explored if PKD1 was activated by detecting both phosphorylation at S744/748 in the kinase activation loop and autophosphorylation at S916 at its C terminus. Cells were infected with HRV16 at a multiplicity of infection (MOI) of 20 for 1 h, followed by an incubation period up to 7 h postinfection (hpi). At various time points postinfection, cells were harvested, and cell extracts were prepared for analysis. The particular MOI and time course protocol were previously optimized and validated to generate synchronous infection (9). Under these conditions, Golgi membrane disruption begins at 3 hpi, and 2C expression begins to be seen at 4 hpi and continues to increase up to 7 hpi, at which point cells are clearly still viable (9). Consistent with data from our previous studies, we confirm here that with this protocol, viral RNA replication peaks at 6 hpi (Fig. 1A), and viral protein expression also peaks at 6 hpi (Fig. 1B to D). Western blotting of HeLa cell extracts from each time point was performed with antibodies to the phospho-S744/S748 activation loop site, which is common to PKD1 and PKD2, and with antibodies specific for the phospho-S916 autocatalytic site on PKD1 (pPKD1 S916) (Fig. 1B). The data revealed PKD1 phosphorylation at both sites starting at between 5 and 6 hpi, clearly when replication was still active. The anti-pPKD S744/748 antibody (pActivation loop) revealed two bands; however, only the upper band changed in intensity following activation by phorbol ester (phorbol 12,13-dibutyrate [PDBu]) treatment, and consequently, we took this to reflect PKD phosphorylation. Uninfected cells (Fig. 1B, lane 1) and cells infected with UV-inactivated virus (lane 9) served as negative controls, and treatment of HeLa cells with PDBu served as a positive control for PKD phosphorylation (lane 2). The UV-inactivated virus control at 7 hpi revealed that PKD activation was clearly dependent upon viral replication. As Toll-like receptor (TLR) agonists have been reported to activate PKD (54), this control excluded the possibility that PKD was being activated by potential TLR agonists present as contaminants in our viral preparation. The time course of viral replication was also controlled in the same experiment by the detection of HRV 2C and 2BC expression with an anti-2C antibody, and it is evident that protein expression does not peak until 6 hpi. In an identical experimental design, PKD2 phosphorylation could also be detected at both sites by using an antibody that detects activation loop phosphorylation as described above and an antibody specific for the phospho-S876 autocatalytic site on PKD2 (pPKD2 S876) (Fig. 1C). To confirm that PKD phosphorylation was not an artifact of HeLa cells, we repeated the study using primary human bronchial epithelial cells (HBECs) (Fig. 1D). Viral replication-dependent activation loop phosphorylation in HBECs followed a time course similar to that in HeLa cells, although induction of PKD1 S916 phosphorylation over the control was less evident. In both cell types, the timing of PKD phosphorylation appeared to coincide with the peak of viral protein expression.

### Chemical inhibitors of PKD block picornavirus replication.

**FIG 2:**
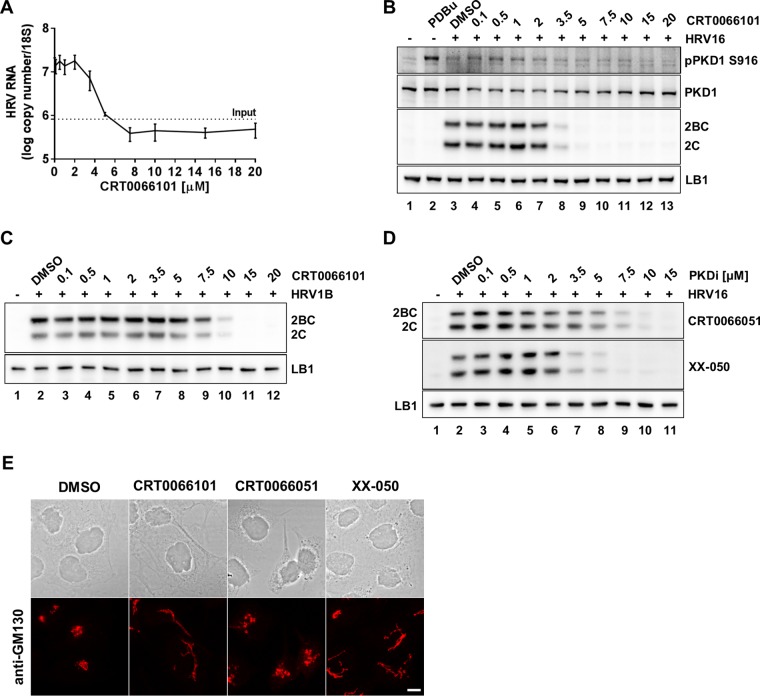
Effect of CRT0066101 on HRV 2C and viral RNA expression following infection. (A) HeLa cells were pretreated for 1 h with increasing concentrations of CRT0066101, followed by infection with HRV16 at an MOI of 20 for 1 h. Following a 6-h replication period, RNA was extracted from cell lysates, and the viral RNA level was quantified by qRT-PCR and normalized to the 18S RNA level. The results show the means (±SEM) from three independent experiments, each performed in duplicate. The “input” level (dotted line) reflects the viral RNA that was cell bound at the start of the replication cycle. (B) HeLa cells were pretreated for 1 h with increasing concentrations of CRT0066101, followed by infection with HRV16 at an MOI of 20 for 1 h. Cell extracts were prepared following a 6-h replication period and analyzed by Western blotting with antibodies to autophosphorylation residue S916 of PKD1, PKD1, HRV 2C, and LB1. Controls are as follows: uninfected cells (lane 1), PDBu-treated cells (lane 2), and vehicle control-treated cells (lane 3). Cells treated with CRT0066101 at concentrations from 0.1 to 20 μM are shown in lanes 4 to 13. (C) HBECs cells were pretreated for 1 h with increasing concentrations of CRT0066101, followed by infection with HRV1B at an MOI of 20 for 1 h. Cell extracts were prepared following a 6-h replication period and analyzed by Western blotting with antibodies against HRV 2C and LB1. Uninfected cells are shown in lane 1, and vehicle control-treated cells are shown in lane 2. (D) The effect of CRT0066051 and XX-50 on HRV 2C protein expression was analyzed by using the same protocol as the one described above for panel B. Results from each experiment shown in panels B to D are representative of data from three independent repeats. (E) In order to confirm the pharmacodynamic effect of the inhibitors on cells, HeLa cells were treated for 8 h with CRT0066101 and XX-050 at 5 μM and CRT0066051 at 10 μM, followed by analysis by confocal microscopy. The Golgi apparatus was revealed by staining with an anti-GM130 antibody, followed by staining with an anti-rabbit antibody coupled to Alexa Fluor 546 (bar = 10 μm).

Having shown that HRV infection appears to activate PKD, we went on to explore the influence of chemical inhibitors of PKD on HRV replication. The compound CRT0066101 is a specific and potent PKD inhibitor that has been used in studies exploring its potential as an anticancer target (48, 51). This inhibitor has a 50% inhibitory concentration (IC_50_) of 1 nM against purified PKD in biochemical assays and a 50% effective concentration (EC_50_) of 1 μM in a cell-based assay (Table 1). We pretreated HeLa cells with increasing concentrations of the drug for 1 h followed by infection with HRV16. Viral replication was subsequently measured at 6 hpi by quantitative real-time PCR (qRT-PCR) (Fig. 2A) and by Western blotting using an antibody against the viral nonstructural protein 2C (Fig. 2B). Figure 2A and B show that CRT0066101 clearly inhibits viral RNA and viral protein expression at concentrations above 3.5 μM. Importantly, we also show that PKD1 phosphorylation induced by viral infection was inhibited by the drug in the same dose-dependent manner by Western blotting with an anti-pPKD1 S916 antibody (Fig. 2B). A similar dose-dependent inhibition of viral protein expression was seen with HBECs infected with HRV1B (Fig. 2C). Furthermore, we tested two novel and structurally different PKD inhibitors (CRT0066051 and XX-050) (Table 1) and confirmed that they too inhibited HRV 2C expression although with different potencies (Fig. 2D). Kinase selectivity profiling of all three PKD inhibitors was performed against a panel of protein and lipid kinases at a drug concentration of 1 μM and at an ATP concentration equivalent to the *K_m_* of each kinase (see Table S1 in the supplemental material). This analysis revealed that in common with most kinase inhibitors, these three PKD inhibitors displayed activity against a number of other protein kinases; however, where these “off-target” inhibitory activities were potentially significant, they did not overlap (Table S1), and there was no significant activity against lipid kinases. Since PKD is known to be involved in regulating the architecture of the Golgi apparatus, we confirmed the pharmacodynamic effect of these inhibitors by demonstrating their ability to remodel the Golgi membrane by confocal microscopy and staining of the *cis*-Golgi matrix protein GM130 (Fig. 2E). All three inhibitors were clearly able to induce morphological changes in the Golgi membrane at the minimal concentration required to block viral replication. These studies clearly and consistently revealed that CRT0066101 was able to completely inhibit viral RNA replication, viral protein expression, and PKD1 S916 phosphorylation at a concentration of 5 μM in HeLa cells.

**TABLE 1:**
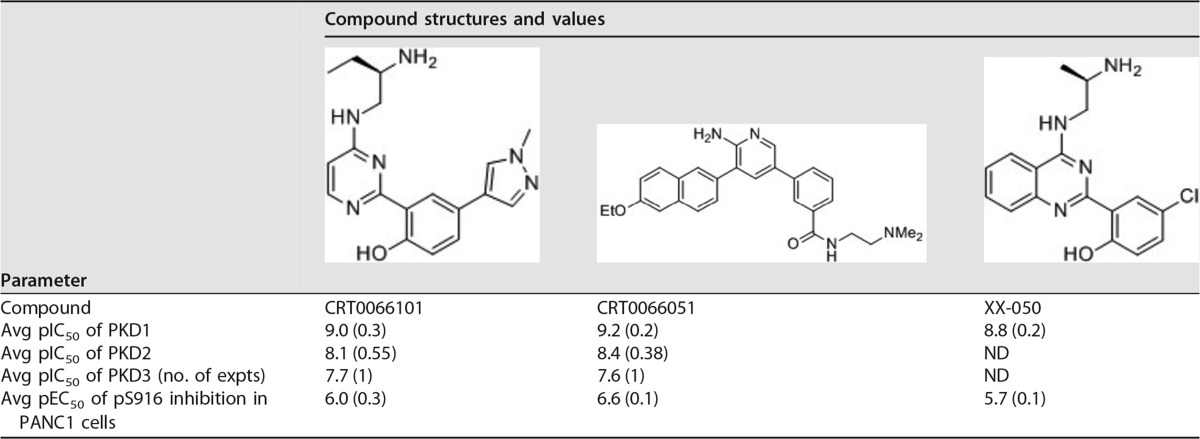
PKD inhibitors^*a*^

a Structures and key pharmacological activities of CRT0066101, CRT0066051, and XX-050 are shown. pIC_50_s for the three inhibitors were determined against purified recombinant kinases in *in vitro* assays as previously described (68, 69). Values are averages of data from at least 2 experiments unless otherwise stated. Standard deviations are shown in parentheses. The pEC_50_ was determined in PANC1 cells by measuring the inhibition of S916 phosphorylation (pS916). Abbreviations: ND, not determined; pIC_50_, −log_10_ value of the molar drug concentration required to give half-maximal inhibition; pEC_50_, −log_10_ value of the molar drug concentration required to give a half-maximal response.

**FIG 3:**
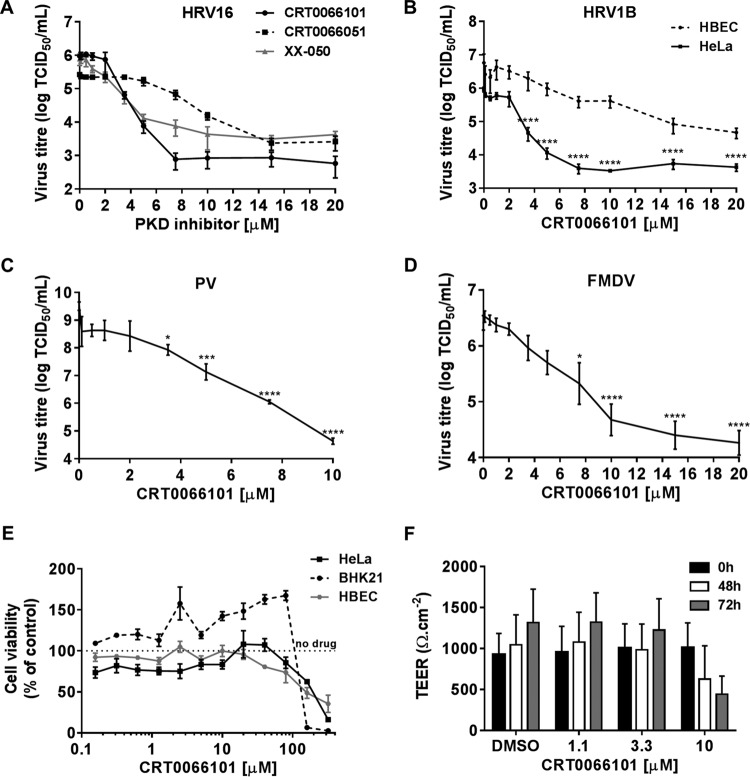
Effect of PKD inhibitors on picornavirus replication. (A) HeLa cells were infected with HRV16 at an MOI of 20, and replication was allowed to proceed for 6 h in the presence of increasing concentrations of CRT0066101, CRT0066051, or XX-050. Viral replication was determined as the endpoint titer (TCID_50_). (B) HeLa cells and HBECs were infected with HRV1B at MOIs of 1 and 5, respectively, and replication was allowed to proceed for 6 h in the presence of increasing concentrations of CRT0066101. Viral replication was determined as the endpoint titer (TCID_50_). (C and D) HeLa cells were infected with PV (C) and BHK21 cells were infected with FMDV (D) at an MOI of 20, replication was allowed to proceed for 6.5 h in the presence of increasing concentrations of CRT0066101, and viral replication was determined as the endpoint titer (TCID_50_). All the virus titer graphs show the means (±SEM) of data from three independent experiments. Differences between infected DMSO-treated cells and drug-treated cells were estimated by using one-way ANOVA with Dunnett's *post hoc* test. *, *P* < 0.05; ***, *P* < 0.001; ****, *P* < 0.0001. (E) HBECs and BHK21 and HeLa cells were incubated with increasing concentrations of CRT0066101 for 10, 8.5, and 8 h, respectively, and cell viability was determined as described above. (F) TEER was measured on HBECs grown in ALI cultures and treated with CRT0066101 at increasing concentrations for 48 and 72 h. Results in panels E and F show the means (±SEM) of data from three independent experiments.

We went on to quantify the effect of CRT0066101, CRT0066051, and XX-050 on the replication of HRV16 by measuring viral titers by the determination of the 50% tissue culture infective dose (TCID_50_) (Fig. 3A). The three different compounds all inhibited viral replication but with different potencies, thus confirming the 2C protein expression and genome replication data shown in Fig. 2A to C. We also tested CRT0066101 against HRV1B and showed that it significantly reduced viral titers in HeLa cells and, to a lesser extent, in primary HBECs (Fig. 3B). We subsequently analyzed the effect of CRT0066101 on other picornaviruses. CRT0066101 effectively inhibited the replication of PV in HeLa cells (Fig. 3C) and FMDV in BHK21 cells (Fig. 3D). Due to the shape of the inhibition curves, we were not able to accurately determine the IC_50_; however, CRT0066101 resulted in a maximum inhibition of the viral endpoint titer of between 2 and 4 logs. To exclude the possibility that this inhibition was due simply to cell death, we determined the cytotoxicity profile of CRT0066101 in HeLa cells, BHK21 cells, and HBECs using the Viral ToxGlo assay and were able to observe cytotoxicity at between 6.5 and 10 h only at concentrations in excess of 100 μM (Fig. 3E). Furthermore, we analyzed longer-term CRT0066101 cytotoxicity using HBECs grown in air-liquid interface (ALI) cultures. Transepithelial electrical resistance (TEER) was measured as a readout of cell viability after treatment with different concentrations of the drug for 48 and 72 h. Cytotoxicity was observed only at a concentration of 10 μM at these time points. Thus, we confirmed that the PKD inhibitor CRT0066101 blocked the replication of HRV16, HRV1B, PV, and FMDV in a number of transformed and primary cells and that there was an acceptable window of separation between efficacy and cytotoxicity. Moreover, PKD inhibitors of diverse chemical structures and selectivities also inhibited HRV16 replication.

### Time-of-addition and endocytosis studies of CRT0066101.

**FIG 4:**
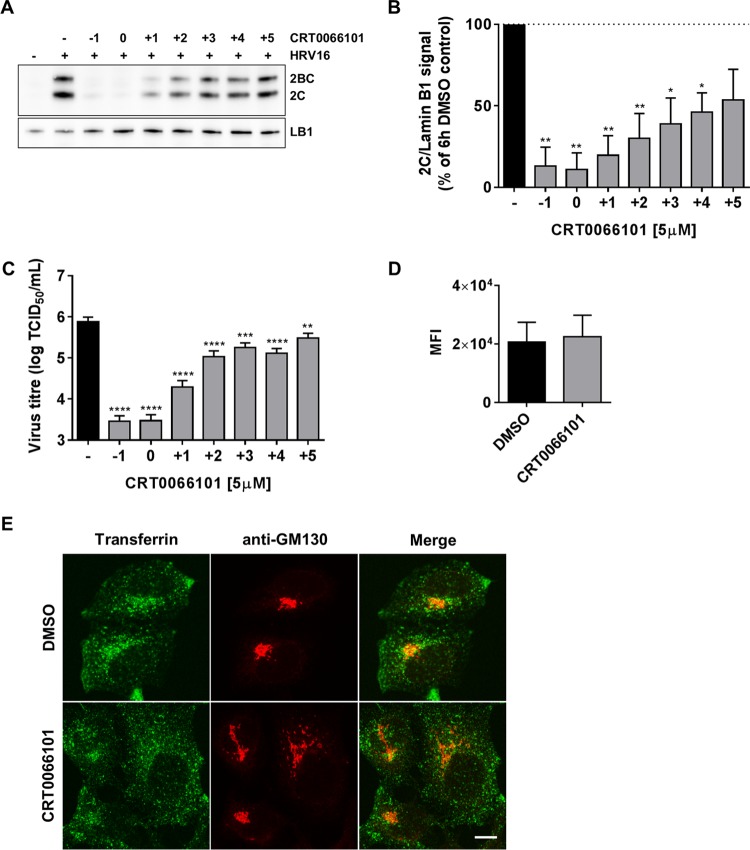
Effect of CRT0066101 on viral entry. (A) HeLa cells were infected with HRV16 (MOI of 20) for 6 h, and CRT0066101 (5 μM) was added at the following different time points: 1 h before infection (−1), during the 1-h virus infection period (0), and every hour after the time of virus adsorption (+1, +2, +3, +4, and +5). Cells extracts were prepared at the end of the 6-h replication period and analyzed by Western blotting with anti-2C and anti-LB1 antibodies. Uninfected cells and DMSO-treated cells infected for 6 h were used as controls. Data from a representative experiment from three independent repeats are shown. (B) 2C Western blots were scanned as described in Materials and Methods and quantified by using ImageJ. The mean 2C/LB1 ratio (±SEM) is shown as a percentage of the value for the DMSO control from three independent experiments. (C) In parallel, virus was extracted from the cell lysates, and viral replication was quantified by endpoint titer determination (TCID_50_). Results are the means (±SEM) of data from three independent experiments, each done in triplicate. Differences between DMSO-treated cells (−) and the rest of the conditions in both panels B and C were estimated by using one-way ANOVA with Dunnett's *post hoc* test. *, *P* < 0.05; **, *P* < 0.01; ***, *P* < 0.001; ****, *P* < 0.0001. (D) HeLa cells were grown on coverslips and pretreated with DMSO or CRT0066101 at 5 μM for 1 h at 37°C. Human transferrin conjugated to Alexa Fluor 488 was added at a 75-μg/ml final concentration, and cells were incubated at 37°C for 1 h in the presence of DMSO or CRT0066101. Cells were stained with an anti-GM130 antibody followed by an anti-mouse antibody coupled to Alexa Fluor 546 and analyzed by confocal microscopy. Transferrin quantification is shown as the mean fluorescence intensity (MFI) from multiple low-power images. For each experiment, images from five different low-power fields with approximately 250 cells per field were quantified by using ImageJ. Data shown are the means (±SEM) from three independent experiments. No statistically significant difference between DMSO- and CRT0066101-treated cells was found by performing a two-tailed *t* test. (E) High-power images of HeLa cells showing internalized transferrin (green) and Golgi membrane (GM130) (red) staining from a representative experiment (bar = 10 μm).

Since CRT0066101 effectively inhibited early events in the viral life cycle and given that PKD is known to be involved in membrane traffic, one possible explanation was that this compound disrupts endocytosis and thus blocks virus entry into cells. To explore if CRT0066101 was blocking viral entry, we performed drug “time-of-addition” studies (Fig. 4A to C). The compound was added to HeLa cells either 1 h prior to viral infection, at the start of the 1-h infection period, or after the infection period every hour up to 5 hpi. At 6 hpi, cells were harvested, and cell extracts were prepared for Western blotting or for endpoint titer determination by a TCID_50_ assay. By both Western blotting of 2C and 2BC expression (Fig. 4A and B) and endpoint titer analysis (Fig. 4C), it is evident that CRT0066101 was able to inhibit HRV16 replication even when added after the infection period, although the efficacy clearly diminished with time. Thus, CRT0066101 is most effective when preincubated with cells or when added to cells during viral infection, but it retains significant inhibitory activity even when added after infection. To further explore whether CRT0066101 blocks clathrin-mediated endocytosis (CME), we examined and quantified the uptake of fluorescently labeled transferrin by HeLa cells in the presence or absence of the drug and found that there was no significant difference between the two conditions (Fig. 4D). In vehicle-treated cells, we observed typical endosomal staining and normal Golgi membrane architecture, whereas in drug-treated cells, endosomal staining appeared normal, while the Golgi membrane was often seen as distended and tubulated, confirming the pharmacodynamic effect of the drug in this particular cell type (Fig. 4E). These studies suggest that the antiviral mechanism of action of CRT0066101 is unlikely to be through the inhibition of viral entry or through an alteration of CME.

### Overexpression of PKD1 mutants.

**FIG 5:**
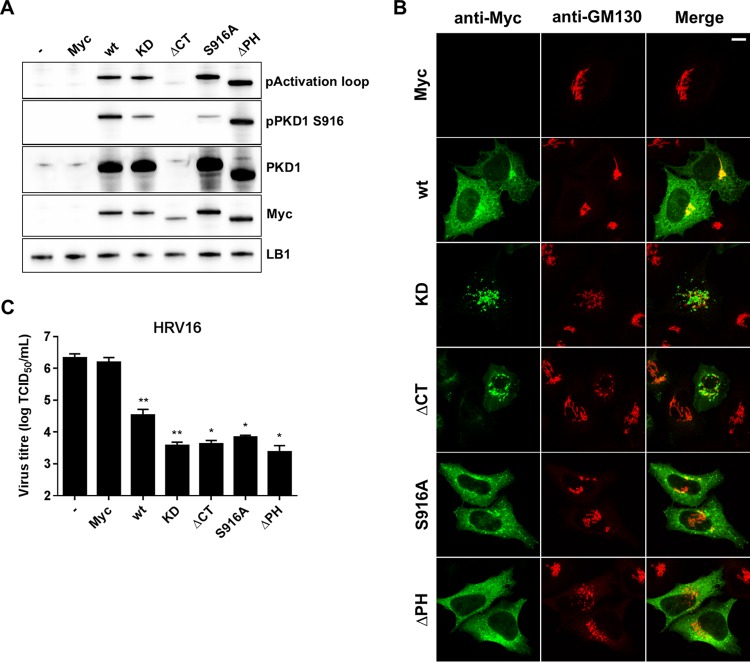
Effect of PKD1 mutant overexpression on HRV16 replication. (A) HeLa cells were transfected with PKD1 plasmids for 18 h, and cells were harvested and processed for Western blotting with specific antibodies against the phosphorylation site in the activation loop (pActivation loop) and autophosphorylation residue S916 of PKD1 and with antibodies to PKD1 and LB1 as loading controls. An anti-Myc antibody was used to check tagged-protein expression. Untransfected cells (−) and empty Myc plasmid-transfected cells (Myc) were used as controls. (B) HeLa cells were transfected as described above for panel A, fixed, immunostained by using an anti-Myc antibody followed by an anti-mouse antibody coupled to Alexa Fluor 488 and with an anti-GM130 antibody followed by an anti-rabbit antibody coupled to Alexa Fluor 546, and analyzed by confocal microscopy (bar = 10 μm). (C) HeLa cells were either untransfected (−) or transfected with each PKD1 construct, incubated for 18 h, and then infected with HRV16 at an MOI of 1 for 1 h, and replication was allowed to proceed for 6 h. Cells were harvested, and cell lysates were processed for endpoint titer determination by a TCID_50_ assay. The graph shows the means (±SEM) of data from 4 experiments for Myc, the wt, and KD and from 3 experiments for ΔCT, S916A, and ΔPH, and each independent experiment was performed in duplicate. Differences between empty Myc plasmid-transfected cells and PKD1 construct-transfected cells were estimated by one-way ANOVA with Dunnett's *post hoc* test. *, *P* < 0.05; **, *P* < 0.01.

In order to investigate the role of PKD1 in HRV replication, we produced a number of Myc-tagged PKD1 wild-type (wt) and mutant cDNA expression vector constructs, including wt PKD1, kinase-dead (KD) PKD1 (K612A), inactive PKD1 lacking the C-terminal region from the kinase domain (ΔCT), inactive PKD1 that is unable to autophosphorylate the autocatalytic residue S916 (S916A), and constitutively active PKD1 lacking the PH domain (ΔPH). First, the different mutants were characterized by Western blotting (Fig. 5A) and confocal microscopy (Fig. 5B). The plasmids were individually transfected in HeLa cells, and after an 18-h incubation period, cells were either harvested and lysed for Western blotting or fixed and immunostained for confocal microscopy. The expression of each transfected PKD1 construct was analyzed by Western blotting with an anti-Myc antibody and phosphorylation of the kinase with antibodies against the activation loop and autophosphorylation residue S916. All constructs expressed protein that migrated at the expected molecular mass, and wt PKD1 was phosphorylated in both the activation loop and the S916 autocatalytic site. The KD mutant showed normal activation loop phosphorylation but reduced S916 autophosphorylation (S916p), whereas the ΔCT mutant showed no S916 phosphorylation and greatly reduced activation loop phosphorylation. The S916A mutant showed slightly enhanced activation loop phosphorylation and a low level of S916p, presumably due to the phosphorylation of endogenous PKD1, and the ΔPH mutant showed enhanced phosphorylation at both sites. Untransfected and empty Myc plasmid-transfected cells were used as controls. The cellular localization of each PKD1 construct was monitored by confocal microscopy with an anti-Myc antibody, and the effect on Golgi membrane morphology was studied by costaining using an anti-GM130 antibody. Different patterns of distribution of wt PKD1 and mutant PKD1 were observed. The expression of wt PKD1 showed both diffuse cytoplasmic staining and strong colocalization with the Golgi membrane, which appeared to have a normal morphology. The KD mutant induced GM130 staining to appear more distended and PKD1 staining to localize exclusively to the Golgi membrane. Similarly, the ΔCT mutant localized to the Golgi membrane and induced a morphological change. The S916A mutant showed no Golgi membrane localization and relatively normal Golgi membrane morphology, whereas the ΔPH mutant also showed no Golgi membrane colocalization but showed substantial Golgi membrane morphological disruption. We then went on to analyze the effect of these mutants on HRV16 replication (Fig. 5C). HeLa cells were transfected with each plasmid and then incubated for 18 h prior to infection with HRV16 at an MOI of 1. Replication was allowed to proceed for 6 h, and cells were then harvested and processed for the determination of endpoint titers (TCID_50_). This protocol was carefully optimized to ensure both a high transfection efficiency (70 to 80%) and consistent and robust viral replication in untransfected and empty vector-transfected cells. Under these conditions, it is clear that the overexpression of either wt PKD1 or any of the mutants significantly decreased viral titers by about 2 logs, whereas control transfection with an empty vector had no effect. Thus, the overexpression of the wt or PKD1 mutants in the context of viral infection has a profound effect, reducing viral replication.

### PKD1 and PKD2 knockout cell lines.

**FIG 6:**
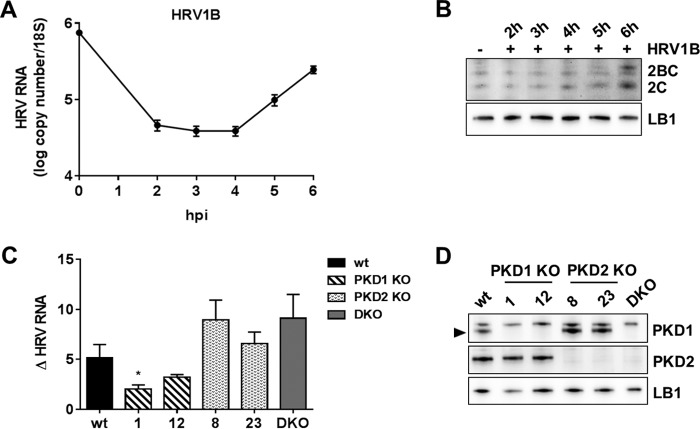
Effect of PKD knockout on HRV replication. (A) Parental wt HAP1 cells were infected with HRV1B at an MOI of 5 for 1 h, and replication was allowed to proceed for up to a 6-h time course. Cells were harvested every hour from 2 to 6 hpi, RNA was extracted from cell lysates, and the viral RNA level was quantified by qRT-PCR and normalized to the 18S RNA level. The 0-h time point corresponds to viral RNA that was cell bound at the start of the replication cycle. Results are shown as means (±SEM) of data from three independent experiments, each performed in duplicate. (B) Parental wt HAP1 cells were infected with HRV1B for 1 h at an MOI of 5, and replication was allowed to proceed for various times up to 6 hpi. Cell extracts were prepared and analyzed by Western blotting with anti-HRV 2C and anti-LB1 antibodies. (C) All clones were infected with HRV1B at an MOI of 5 for 1 h, followed by a replication period of up to 6 h. Viral RNA was extracted and quantified by qRT-PCR, the level was normalized to the total cellular 18S RNA level, and the fold increase in the viral RNA level from 3 to 6 hpi was calculated for each clone (Δ HRV RNA). Results are the means (±SEM) from four independent experiments, each performed in duplicate. Differences between parental wt HAP1 and knockout cells were estimated by one-way ANOVA with a two-tailed *t* test *post hoc* analysis. *, *P* < 0.05. The fold increase was statistically different between wt cells and clone 001 (*P* = 0.0488). (D) Cell extracts were prepared from the parental wt HAP1 clone (wt) (lane 1), PKD1 knockout clones (clones 1 and 12) (lanes 2 and 3), PKD2 knockout clones (clones 8 and 23) (lanes 4 and 5), and a double-knockout clone (DKO) (lane 6) and analyzed by Western blotting with anti-PKD1- and anti-PKD2-specific antibodies and an anti-LB1 loading control. The PKD1-specific band is shown by an arrowhead.

HAP1 cell lines in which PKD1 or PKD2 genes had been knocked out by clustered regularly interspaced short palindromic repeats (CRISPR)-Cas9 were obtained, and a double knockout (DKO) was generated as described in Materials and Methods. We were unable to infect these cells with HRV16; however, HRV1B was able to replicate albeit very weakly. We performed a viral replication time course in parental wt HAP1 cells with HRV1B at an MOI of 5 (Fig. 6A). We measured viral RNA levels by qRT-PCR and showed that there is an initial phase of net viral RNA degradation, similarly to infection in HeLa cells (Fig. 1A), followed by active RNA replication. However, viral RNA levels never reached the input level (viral RNA that was cell bound at the start of the replication cycle), showing the capacity of these cells to degrade viral RNA. We also confirmed that viral protein was being made by checking HRV 2C and 2BC expression levels by Western blotting, although we were able to detect only a low level of expression at 6 hpi (Fig. 6B). To assess differences in viral replication among the various clones, each clone was infected with HRV1B for 1 h, followed by incubation periods up to 6 hpi. Viral genome replication was quantified by qRT-PCR, and the fold increase in viral RNA levels from 3 to 6 hpi was determined (Fig. 6C). The results demonstrated that the rate of viral genome replication was reduced in the PKD1 knockout clones compared to the parental wt cell line, but there was a trend toward increased replication in the PKD2 and DKO clones. Western blotting of parental wt cells, two independent PKD1 clones, two independent PKD2 clones, and the DKO with specific antibodies to PKD1 and PKD2 confirmed that the parental cells expressed both PKD1 and PKD2 and that in the respective knockouts, there was no detectable protein expression (Fig. 6D).

### PKD inhibitors do not act by enhancing interferon signaling.

**FIG 7:**
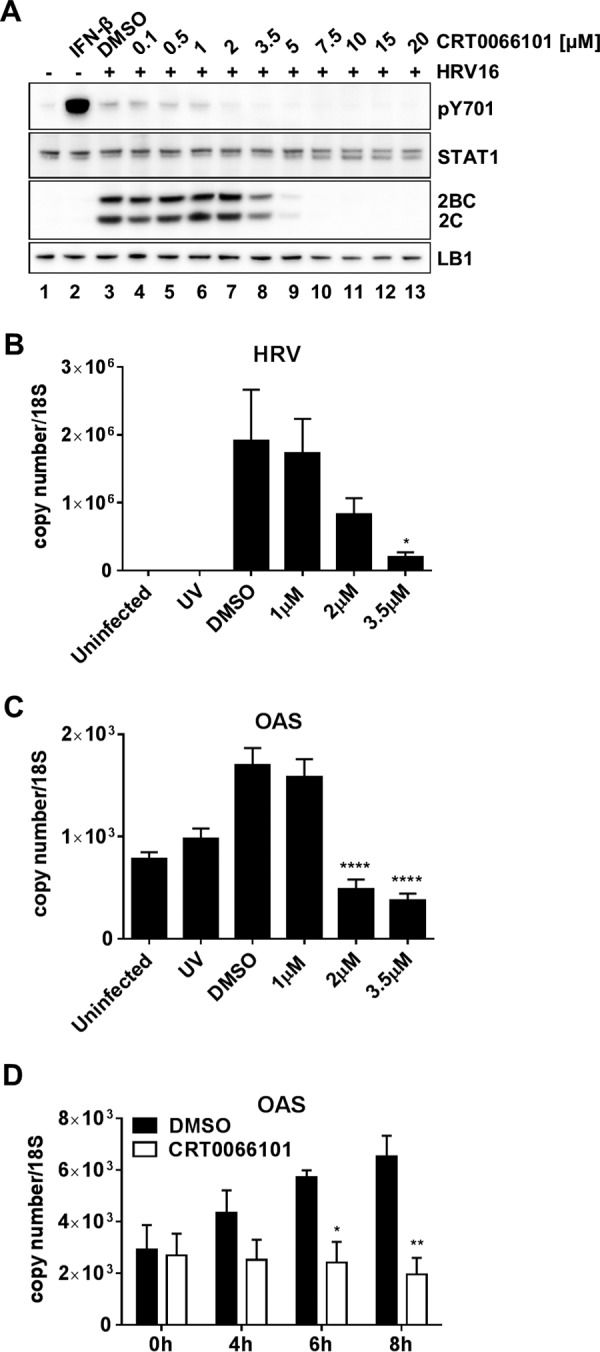
Effect of CRT0066101 on interferon signaling. (A) Effect of CRT0066101 on STAT1 phosphorylation at residue Y701 in HeLa cells infected with HRV16. Cells were either untreated (lane 1) or treated with 30 U/ml IFN-β for 15 min (lane 2), the DMSO vehicle (lane 3), or increasing concentrations of CRT0066101 for 1 h, followed by a 6-h replication period. Cell extracts were prepared and analyzed by Western blotting with antibodies to pSTAT1 Y701, STAT1, HRV 2C, and LB1. Data shown are representative of results from three independent experiments. (B and C) To determine the effect of CRT0066101 on ISG expression, RNA was extracted from HRV16-infected HeLa cells after 20 h of culture in the presence of the DMSO vehicle or 1 μM, 2 μM, or 3.5 μM CRT0066101. UV-inactivated virus was included as a control. Viral replication was confirmed by measuring the levels of HRV16 RNA (HRV) (B) and OAS mRNA (C) as a representative ISG. The results are the means (±SEM) of data from four independent experiments, each performed in duplicate. Differences between infected DMSO-treated cells and infected CRT0066101-treated cells were determined by one-way ANOVA with Dunnett's *post hoc* analysis. *, *P* < 0.05; ****, *P* < 0.0001. (D) HeLa cells were pretreated for 1 h with the DMSO vehicle or CRT0066101 at 5 μM, followed by stimulation with IFN-β (30 U/ml) for 4, 6, and 8 h in the presence of DMSO or CRT0066101. Cells were harvested, and RNA was extracted and processed for qRT-PCR. The OAS mRNA level was measured and normalized to the 18S RNA level. The results are the means (±SEM) of data from three independent experiments, each performed in duplicate. Differences between DMSO-treated and CRT0066101-treated cells at each time point were determined by two-way ANOVA with Sidak's *post hoc* test. *, *P* < 0.05; **, *P* < 0.01.

It was previously shown that PKD activation suppresses type I IFN signaling by phosphorylating IFNAR and causing receptor downregulation. It was therefore postulated that PKD inhibitors would be antiviral by enhancing type I IFN signaling (52, 53). To test this hypothesis, we infected HeLa cells with HRV16 at an MOI of 20 in the presence of increasing concentrations of CRT0066101 and allowed replication to proceed for 6 h. Cell extracts were analyzed by Western blotting, and activation of the IFNAR signaling pathway was revealed by immunodetection of STAT1 phosphorylated at Y701 (pY701) (Fig. 7A). If the antiviral activity of CRT0066101 was acting by enhancing IFNAR signaling, one would expect to see enhanced STAT1 phosphorylation following HeLa cell infection in the presence of the drug. However, we observed that during viral infection and the viral replication cycle, CRT0066101 inhibits pSTAT1 levels in a concentration-dependent manner. The cellular IFN response to viral infection was clearly not maximal, as we were able to generate a much stronger pSTAT1 response by adding exogenous IFN-β; therefore, had CRT0066101 been acting by enhancing IFN signaling, we would have expected to see this response. To further confirm that CRT0066101 did not enhance the IFN response, we examined the induction of 2′-5-oligoadenylate synthetase (OAS), as a marker of interferon-stimulated gene (ISG) induction, in HeLa cells infected with HRV16 in the presence of CRT0066101 20 h following infection with live virus. Longer infection times than those used in previous experiments were required to see significant OAS induction. We confirmed that viral RNA levels were increased significantly compared to viral RNA replication in UV-inactivated-virus-infected cells, and viral RNA replication was effectively inhibited by treatment with increasing concentrations of CRT0066101 (Fig. 7B). OAS mRNA was induced by infection with live virus and was significantly inhibited by treatment with the drug (Fig. 7C). Thus, it was clear that CRT0066101 inhibited STAT1 phosphorylation and ISG induction, and there was no evidence to suggest that CRT0066101 enhanced IFN signaling in virally infected cells. Furthermore, we tested the effect of CRT0066101 on type I IFN signaling by measuring OAS mRNA levels during a time course of IFN-β stimulation (Fig. 7C). HeLa cells were pretreated for 1 h with the dimethyl sulfoxide (DMSO) vehicle or CRT0066101 at 5 μM followed by stimulation with IFN-β for 4, 6, and 8 h in the presence of DMSO or the drug. The OAS mRNA level was significantly reduced in CRT0066101-treated cells compared to DMSO-treated cells at 6 and 8 h poststimulation. OAS mRNA remained at a basal level in CRT0066101-treated cells, whereas it followed the expected increase in a dose-dependent manner after IFN-β stimulation in DMSO-treated cells, confirming that CRT0066101 does not act through enhancing type I IFN signaling.

**FIG 8:**
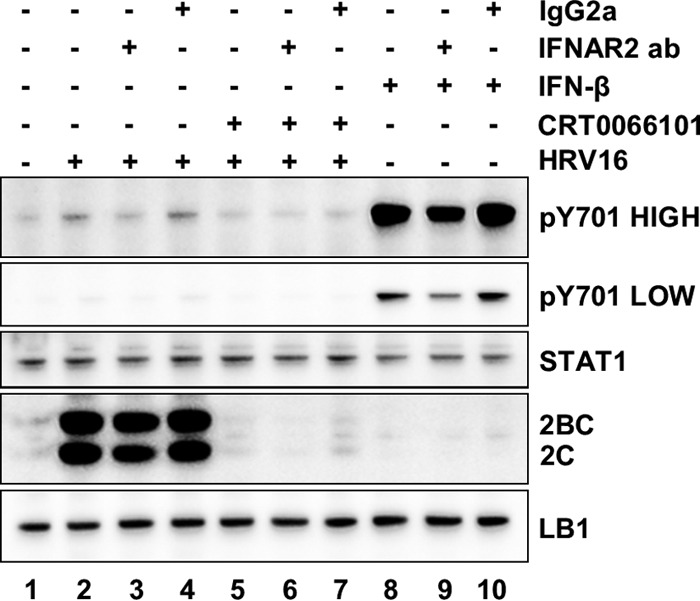
Effect of blocking of type I interferon receptor signaling on CRT0066101. In order to determine if a blockade of the type I interferon receptor (IFNAR2) influenced the ability of CRT0066101 to inhibit viral replication, HeLa cells were left untreated (lane 1) or pretreated with DMSO (lanes 2 to 4) or 5 μM CRT0066101 (lane 5 to 7) for 1 h. Cells were subsequently infected with HRV16 for 1 h (lanes 2 to 7), and replication was allowed to proceed for a further 4 h. During the viral infection and replication periods, cells were treated with a blocking antibody to IFNAR2 alone (lane 3) or with an isotype-matched control antibody alone (lane 4) or cotreated with CRT0066101 and a blocking antibody (lane 6) or CRT0066101 with an isotype control (lane 7). As additional controls, cells were treated with 30 U/ml of IFN-β for 4 h (lanes 8 to 10), with no antibody (lane 8), with anti-IFNAR2 (lane 9), or with an isotype control antibody (lane 10). Cell extracts were prepared and analyzed by Western blotting with antibodies to pSTAT1 Y701, STAT1, HRV 2C, and LB1. The pSTAT1 Y701 blot is shown at high and low exposures, and data shown are from a representative experiment from three independent repeats.

In order to explore further if the mechanism of action of CRT0066101 was through enhanced IFN signaling, we tested if the antiviral effect of the drug could be blocked or reduced by coadministration of the drug with an antibody that blocked IFNAR function. If the drug mechanism was through enhancing the antiviral state induced by autocrine stimulation by type I IFN, the antiviral effect of CRT0066101 would be suppressed by a blocking anti-IFNAR antibody. By using the phosphorylation of STAT1 at Y701 as a readout for type I IFN signaling, cell extracts were examined 4 h following infection of HeLa cells with HRV16. Viral replication was confirmed by 2C and 2BC expression, and this corresponded with enhanced STAT1 phosphorylation (Fig. 8, lanes 1 and 2). The phosphorylation of STAT1 in infected cells was partially inhibited by the blocking IFNAR antibody (Fig. 8, lane 3) but not the isotype-matched control antibody (lane 4). The control antibody and the anti-IFNAR antibodies neither inhibited nor enhanced viral replication, as revealed by 2C and 2BC expression. As described above, CRT0066101 at 5 μM completely blocked 2C and 2BC expression (Fig. 8, lane 5). However, this inhibition of viral replication by the drug was not reversed by either the blocking IFNAR antibody or the control antibody (lanes 6 and 7). As an antibody control, uninfected HeLa cells were stimulated with IFN-β, which produced a robust pSTAT1 signal (Fig. 8, lane 8) that was reduced by the inhibitory anti-IFNAR antibody (lane 9) but not by the isotype-matched control antibody (lane 10). As IFN stimulation caused much greater STAT1 phosphorylation than did viral infection, the same blot is shown with both high and low levels of exposure. Thus, suppression of signaling through IFNAR with a blocking antibody does not diminish the efficacy of CRT0066101 in inhibiting viral replication.

## DISCUSSION

Previous studies have shown that during their replication cycle, picornaviruses remodel cellular ER and Golgi membranes, and PI4KB, OSBP, and GBF1/Arf1 have been identified as important host factors in the viral replication process (10–27). Since PKD interacts with all of these proteins (34, 36–40) and PKD is known to regulate Golgi membrane morphology, vesicle trafficking, and lipid homeostasis, we hypothesized that PKD may also be involved in viral replication.

We first examined if HRV16 activated PKD by analyzing cell lysates from virally infected HeLa cells and HBECs. Using antibodies that specifically recognize PKD1 and PKD2 phosphorylated in their activation loop at S744/748 (indicative of the activation of the kinase by upstream effectors such as PKCε) and the autophosphorylation of PKD1 at S916 and of PKD2 at S876, it was clear that viral infection induced the phosphorylation of PKD1 and -2 at both sites in a highly reproducible manner in HeLa cells. PKD1/2 phosphorylation at S744/748 was also detected in HBECs, although we were unable to reproducibly show S916 phosphorylation. We were able to detect PKD phosphorylation at 5 to 6 hpi, which may indicate that this is a relatively late event in the replication cycle, or it may simply be due to the limit of detection of this event by Western blotting with these particular antibodies. However, the timing of PKD phosphorylation coincides with the peak of viral protein expression and occurs while RNA genome replication is still active and prior to the cytopathic effect (CPE). Since PKD can be activated by caspase cleavage during apoptosis (46), this is clearly a potential mechanism that might be involved late in the replication cycle. However, we have never seen a PKD cleavage product by Western blotting in HeLa cell extracts that would suggest that this was occurring (data not shown). In order to examine if this activation of PKD was important for the viral life cycle, we tested a well-validated chemical inhibitor of PKD, namely, CRT0066101, which has previously been studied as a potential cancer chemotherapeutic agent and was well tolerated at doses up to 80 mg/kg of body weight in animal models (48, 51). We have shown that this PKD inhibitor was able to completely inhibit HRV 2C and 2BC protein expression and viral RNA replication in HRV16-infected HeLa cells at 5 μM and was also able to inhibit virally induced PKD1 S916 phosphorylation at this concentration. This is a concentration well below that required for the cytotoxic effect of the compound and a concentration at which the compound clearly has a pharmacodynamic effect on cells, as revealed by changes in the morphology of the Golgi apparatus. Therefore, CRT0066101 appears to shut off viral protein and RNA syntheses, which are clearly early events in the viral replication cycle. We also went on to show that CRT0066101 inhibited the replication of HRV16, HRV1B, PV, and FMDV by viral endpoint titration with reductions in TCID_50_ values of between 2 and 4 logs over the concentration ranges tested. The inhibition of FMDV replication was particularly interesting, as this virus is not thought to have a dependency on PI4KB (28, 29). It is possible that this antiviral effect is due to some off-target effect of CRT0066101, so in order to test this possibility, we examined two other novel and structurally diverse PKD inhibitors in an assay measuring viral protein expression and in a viral endpoint titration assay for HRV16-infected HeLa cells. Although none of the inhibitors are totally specific for PKD, their protein kinase-inhibitory profiles do not overlap, making it highly unlikely that their ability to block viral replication was due to some common off-target activity. All three inhibitors had a >1,000-fold selectivity window over a panel of lipid kinases when tested in an *in vitro* kinase assay, including phosphatidylinositol 4-kinase III alpha (PI4KA) and PI4KB. Further supporting the evidence that the antiviral activity of CRT0066101 is not simply through the inhibition of PI4KB is the observation that this compound also inhibits FMDV replication, yet it is known that this virus is not sensitive to PI4KB inhibitors (28, 29). All three PKD inhibitors clearly blocked viral replication albeit with different potencies and efficacies, which does not correlate with their potency as PKD inhibitors in *in vitro* kinase assays. Although we currently have no clear explanation for this potential discrepancy, one must be cautious not to extrapolate compound potency from *in vitro* kinase assays to cell-based assays. A drop in potency for kinase inhibitors between biochemical assays and cell-based assays is expected and very well documented (55), although for these particular PKD inhibitors, this is at the high end of the observed spectrum (IC_50_ of ∼1 nM and EC_50_ of ∼1 μM). However, PKD is particularly complex, as it has been shown to adopt conformational states that can be stabilized by inhibitors that paradoxically lead to cellular redistribution and enhanced phosphorylation states (56). We have therefore shown that the antiviral effect of PKD inhibitors is not restricted to one cell type, one inhibitor chemotype, or one picornavirus, thus adding significant confidence to our observation for a role for PKD in the replication of certain picornaviruses.

As a third line of evidence, we generated a set of PKD1 mutants and tested their effect on HRV16 replication by measuring viral endpoint titers. First, we characterized their expression and phosphorylation phenotypes by Western blotting, and the results were largely consistent with expectations from previous studies (57). We also examined their effect on Golgi membrane morphology and cellular localization by confocal microscopy. The KD, ΔCT, and ΔPH mutants seemed to cause Golgi membrane morphological changes, which has not previously been described for the ΔCT mutant, and the KD and ΔCT mutants appeared to localize exclusively to the Golgi membrane, with no cytoplasmic staining. Conversely, the S916A and ΔPH mutants appeared to lose their Golgi membrane localization. Second, we analyzed the effect of overexpressing these mutants in the context of viral replication and observed that the overexpression of either wt PKD1 or inactive (KD, S916A, and ΔCT) or superactive (ΔPH) mutants reduced HRV16 titers by about 2 logs, thus confirming the importance of PKD1 for HRV replication.

As a fourth line of evidence, we also went on to test HRV replication in PKD1 knockout HAP1 cells and showed that genome replication is significantly decreased compared to that in the isogenic parental cell line. Interestingly, PKD2 and DKO cell lines showed the opposite effect, suggesting that PKD1 and PKD2 may have differential and indeed opposing effects on viral replication. It is known that PKD1 and -2 can form homo- and heterodimers (58); thus, it is possible that the PKD1 and PKD2 knockouts change the composition of the dimers and, thus, their function. It is challenging at this stage to speculate on the phenotypic differences between PKD1 and PKD2 knockouts and to make direct comparisons with chemical inhibitors, as kinase inhibitors and kinase knockouts do not always phenocopy (59). However, with regard PKD as an antiviral target, these knockout data suggest that one may need a PKD1-selective inhibitor.

These overexpression and knockout studies clearly point to a role for PKD in HRV replication, although they do not define precisely a single molecular target or mechanism of action. This is likely to be challenging, as it may involve complexes of PKD1 and -2 and other interacting proteins and may not depend on a single downstream target. It is known that PKD can both activate PI4KB and inhibit OSBP and CERT, thus acting as a key regulator of lipid transport in the ER and Golgi membranes. Therefore, an inactive PKD mutant could be inhibiting PI4KB, and a superactive PKD mutant could be inhibiting OSBP and CERT; both effects would inhibit viral replication. This would explain why two functionally opposite mutants could inhibit viral replication.

In order to exclude potential mechanisms through which PKD inhibitors blocked picornaviral replication, we examined the possibility that PKD was involved in influencing IFN signaling, as was proposed previously (52, 53). In this model, activated PKD caused the downregulation of IFNAR through phosphorylation and induced degradation, thus suppressing IFN signaling. Therefore, a PKD inhibitor would be antiviral by blocking this suppression of IFN signaling. If this were the case, we would predict that CRT0066101 would enhance IFNAR signaling during viral infection. However, in an assay of HeLa cell infection and replication, we were unable to show that the coadministration of CRT0066101 enhanced IFNAR signaling, as revealed by STAT1 phosphorylation or by the induction of mRNA expression of the IFN-stimulated gene OAS. Indeed, the exact opposite was observed. Moreover, the OAS mRNA level was significantly reduced in CRT0066101-treated cells compared to DMSO-treated cells during a time course of IFN-β stimulation. We were also unable to show that the suppression of IFNAR signaling with a blocking antibody reduced the effectiveness of CRT0066101 as an antiviral agent. All these lines of evidence suggest that the antiviral mechanism of CRT0066101 is not through enhancing autocrine IFN signaling.

PKD clearly has an important role in regulating the function of the TGN, which is a crucial sorting hub for membrane traffic on biosynthetic pathways but is also involved in sorting events for recycling endosomes (60). Thus, agents such as PKD inhibitors that perturb the function of the TGN may also have an impact on recycling endosomes. Although viral entry mechanisms are still relatively poorly characterized, there appears to be good evidence that HRV entry is largely dependent on CME in HeLa cells, particularly for the minor group of rhinoviruses that use the low-density lipoprotein receptor (LDLR) (61, 62) and FMDV, which also enters cells via this pathway (63). CME also appears to be essential for replication due to a requirement for cholesterol trafficking from the plasma membrane to replication organelles (64). We have shown that at a concentration of CRT0066101 that clearly caused morphological changes of the Golgi membrane architecture and was antiviral (5 μM), the uptake of transferrin as a marker of CME appeared qualitatively and quantitatively the same in both DMSO- and CRT0066101-treated cells. Thus, it is unlikely that the drug's antiviral mechanism is through an inhibition of CME. Further evidence in support of this came from time-of-addition studies with CRT0066101. The addition of the drug before infection and during the infection period was most effective at blocking viral replication; however, CRT0066101 still produced significant inhibition even when administered after the end of the infection period, although this effect clearly decreased with time postinfection. This was evident by measuring both 2C expression in cell extracts and viral titers.

In conclusion, we showed first that HRV infection resulted in PKD phosphorylation and second that PKD inhibitors effectively blocked viral protein and RNA expression and consequently blocked the replication of HRV, PV, and FMDV. Our observations were not restricted to one virus or one cell type and were also not restricted to one chemotype of PKD inhibitor, thus giving substantial confidence to the observation. The findings for chemical inhibitors were further supported by two further lines of evidence from PKD1 overexpression and PKD knockout studies. The mechanism of action does not appear to be due to an inhibition of clathrin-mediated endocytosis, nor is it as simple as the inhibition of the downstream effector PI4KB, since CRT0066101 also inhibits the replication of FMDV, which is known not to require this lipid kinase and is not sensitive to PI4KB inhibitors. We were unable to find evidence that supports the hypothesis that PKD inhibition augments the antiviral state of the cell by enhancing IFN signaling. Important questions remain to be answered, such as what activates PKD and at what part of the replication process is PKD required. However, based on our current findings, we propose that PKD1 potentially represents a novel antirhinoviral drug target.

## MATERIALS AND METHODS

### Reagents.

The following primary antibodies were used for Western blotting: rabbit anti-pPKD1 S744/S748 (pPKD1/2 activation loop), rabbit anti-pPKD1 S916, rabbit anti-PKD1, rabbit anti-PKD2, anti-pSTAT1 Y701, rabbit anti-STAT1 (Cell Signaling), rabbit anti-lamin B1 (LB1) (Proteintech), and rabbit anti-pPKD2 S876 (Millipore). The rabbit antibody to HRV 2C was generated and used as previously described (9). Secondary antibodies conjugated to horseradish peroxidase (HRP) were obtained from Jackson ImmunoResearch and were revealed by using the ECL reagent (Geneflow). PDBu) (Sigma) was dissolved in DMSO and used at a final concentration of 200 nM in all the experiments as a positive control for PKD phosphorylation. For the interferon receptor (IFNAR)-blocking antibody studies, either a mouse anti-IFNAR2 antibody (Stratech) or isotype control mouse IgG2a (Abcam) was used at a 5-μg/ml final concentration. Recombinant human IFN-β1α (IFN-β; R&D) was used at a final concentration of 30 U/ml. The primary antibodies used for confocal microscopy were mouse anti-Myc (Merck Millipore) and rabbit anti-GM130 (BD Pharmingen) in combination with a donkey anti-rabbit secondary antibody coupled to Alexa 546 and a donkey anti-mouse secondary antibody coupled to Alexa 488, respectively (Jackson ImmunoResearch).

### Cell culture and viral infection.

The human cervical epithelial cell lines HeLa Ohio (European Collection of Authenticated Cell Cultures [ECACC] 930021013) and HeLa H1 (ATCC CRL-1958) were grown in Dulbecco's modified Eagle's medium (DMEM) supplemented with 1% glutamine, 7.5% sodium bicarbonate, 25 mM HEPES, and 10% fetal bovine serum (FBS). The hamster kidney fibroblast cell line BHK21 (ATCC CCL-10) was grown in Eagle's minimum essential medium (EMEM) (ATCC 30-2003) supplemented with 10% FBS. HBECs (catalog no. CC2540; Lonza) were cultured in bronchial epithelial cell growth medium according to the manufacturer's recommendations (catalog no. CC3170; Lonza).

HRV16 stocks (ATCC VR-283) were produced by infection of HeLa H1 cells and were titrated on HeLa Ohio cells to determine the TCID_50_ per milliliter. For the HRV infection experiments, cells were infected with either HRV16 or HRV1B at the indicated times and MOIs. Infections were synchronized by incubating the virus for 1 h at room temperature (RT), followed by a wash with phosphate-buffered saline (PBS) and the addition of fresh medium before the cells were incubated at 37°C for different times. Infection experiments with PV (type 1 Mahoney strain) and FMDV (type O1K strain) (65, 66) were performed according to the same protocol as the one described above for HRV.

The protocol to measure TEER on HBECs during CRT0066101 treatment was carried out as follows: HBECs from the Cell Culture Core at the University of North Carolina Cystic Fibrosis Center or from Lonza were cultured, as previously described (67), on 12-well clear transwell inserts (0.4-μm pore size). The HBECs from three independent donors developed a well-differentiated mucociliary phenotype when cultured at low passage (passage 3 or earlier [≤P3]) for ≥14 days at the ALI. On day 14 of culture at the ALI, 0.75 ml DMEM (37°C) was added to the apical surface of the inserts, and TEER was measured by using an Evom voltmeter with STX2 chopstick electrodes (World Precision Instruments, UK). Three recordings were made for each insert before DMEM was aspirated from the apical surface to reestablish the ALI. Treatment then commenced with either CRT0066101 (1.1, 3.3, or 10 μM) or the vehicle (0.1% sterile water) added to the basolateral medium. The TEER measurements were repeated 48 and 72 h following the start of treatment with the compound, with medium containing the compound/vehicle being refreshed at 48 h. Group sizes of 3 inserts were used for each treatment, and the mean absolute values for TEER were calculated for each independent donor. Primary HBECs at passage 1 were purchased from S. Randell at the Marsico Lung Institute/Cystic Fibrosis Research Center at the University of North Carolina, Chapel Hill. Human lungs unsuitable for transplantation were obtained under protocol no. 03-1396 approved by the University of North Carolina at Chapel Hill Biomedical Institutional Review Board. Informed consent was obtained from authorized representatives of all organ donors. Primary HBECs purchased from Lonza were obtained under established ethical practices. Informed consent and legal authorization were as defined by Lonza in its product specifications.

### Western blotting.

Following viral infection and replication for various times, cells were lysed in ice-cold radioimmunoprecipitation assay (RIPA) buffer (Sigma) supplemented with protease (Roche) and phosphatase (Sigma) inhibitors (according to the manufacturers' instructions), and their protein content was measured by the bicinchoninic acid assay (Thermo Scientific). Equal amounts of protein were loaded onto 4 to 12% Bis-Tris SDS-PAGE gels (Life Technologies), followed by transfer onto polyvinylidene difluoride (PVDF) membranes (Life Technologies). Membranes were blocked in Tris-buffered saline (TBS) supplemented with 5% bovine serum albumin (BSA) and 0.1% Tween 20 for 1 h at RT.

Primary antibodies were incubated overnight at 4°C, and secondary antibodies were incubated for 1 h at RT, followed by the addition of ECL reagent and data collection on a Fusion FX7 image analyzer (Vilber Lourmat). Analysis of quantified images was performed by using ImageJ.

### PKD knockout HAP1 cells.

PKD knockout cells were engineered by using CRISPR-Cas9 to insert a variable number of base pairs into exon 2, causing a frameshift. Cells were grown in Iscove's modified Dulbecco's medium (IMDM) supplemented with 10% FBS and 1% penicillin-streptomycin. The cells were purchased from Horizon Genomics (PKD1 knockout lines HZGHC000394c001 and HZGHC000394c012 and PKD2 knockouts lines HZGHC000169c008 and HZGHC000169c023). The PKD1/2 DKO cell line was generated with a 139-bp insertion in exon 2 of PKD1 and an 11-bp deletion in exon 2 of PKD2 with the following primers: PKD1 forward primer 5′-TTGTTTCCCTTTTTCATGTGGACAG-3′, PKD1 reverse primer 5′-TTGTATTTGCAGTTCCCTGAATGTG-3′, PKD2 forward primer 5′-CCCATTATTACTTCCTAGGCTGCG-3′, and PKD2 reverse primer 5′-ATCCACCCCTATTTTCCGCCTA-3′. Both the insertion in PKD1 and the deletion in PKD2 caused a frameshift, which led to the PKD1/2 DKO cell line.

To assess viral replication, PKD knockout cells were infected for 1 h with HRV1B at an MOI of 5, followed by incubation at 37°C for the indicated hours postinfection. At each time point, cell extracts were prepared for either Western blotting or RNA extraction and gene expression analysis. Cells were lysed with RLT buffer (Qiagen) supplemented with β-mercaptoethanol (Sigma) at a 1:200 dilution. mRNA extraction was performed by using the RNeasy minikit (Qiagen) according to the manufacturer's instructions. One microgram of mRNA was reverse transcribed for cDNA synthesis for 1 h at 37°C by using the Omniscript RT kit (Qiagen). Quantification of HRV and 18S RNA levels was conducted by using specific primers, and the fold increase in viral RNA levels at between 3 and 6 hpi was calculated. Analysis at each time point was performed in duplicate, and the results shown represent the means (± standard errors of the means [SEM]) from 4 independent experiments. For the Western blot analyses, cells were lysed with RIPA buffer as described above and revealed with antibodies to PKD1, PKD2, HRV 2C, and LB1.

### PKD inhibitors.

CRT0066101 was described previously (48) and was purchased for this study from Tocris, with a purity of 99.9%. CRT0066101 (XX-209) and compound XX-050 were synthesized as previously described (68). CRT0066051 (Y-095) was also synthesized as previously described (69). XX-50 and CRT0066051 preparations had a purity of 100% as determined by liquid chromatography-mass spectrometry (LCMS) and had the correct molecular weight. CRT0066101 can also be obtained in larger quantities from Ximbio. Inhibitors were tested in *in vitro* PKD kinase assays and cellular assays on PANC1 cells as previously described (48). The selectivity of the three PKD inhibitors was determined against a panel of protein and lipid kinases by Eurofins and the MRC PPU International Centre for Kinase Profiling (http://www.kinase-screen.mrc.ac.uk), respectively. Each inhibitor was tested at a single concentration of 1 μM and at an ATP concentration equivalent to the *K_m_* for each kinase.

In order to assay the effect of PKD inhibitors on viral replication, cells were pretreated with increasing concentrations of different PKD inhibitors or the DMSO vehicle alone for 1 h at 37°C. In all cases, the final concentration of the DMSO vehicle in each well was 0.2%. Virus was then added to the cells at MOIs ranging from 1 to 20; incubated for 1 h at RT, followed by washing with PBS to remove unbound virus; and further incubated in fresh medium containing the inhibitor compound or the DMSO vehicle for 6 h at 37°C. At the end of the infection/replication period, cells and the supernatant were scraped, frozen-thawed twice, and centrifuged at 10,000 × g for 5 min at 4°C to remove cell debris, and the supernatant containing the viral particles was used to perform TCID_50_ titration assays. The cytotoxicity of the compounds was determined by using the Viral ToxGlo assay (Promega) according to the manufacturer's instructions. For analysis of cellular and viral proteins by Western blotting, cells were lysed in ice-cold RIPA buffer at the end of the infection/replication period as described below.

### Viral endpoint titer determination (TCID_50_).

HeLa Ohio cells were incubated in 96-well plates in DMEM (supplemented with 2% FBS and 1% penicillin-streptomycin) with 8-fold dilutions of the virus in six replicates for 5 days. Titration was assessed by the presence or absence of CPE in each well by using an HRV16 stock as a positive control.

### Time-of-addition studies.

Cells were infected with HRV16 (MOI of 20) for 6 h, and CRT0066101 (5 μM) was added at the following different time points: 1 h before infection (−1), at the time of virus adsorption (0), and every hour after time zero (+1, +2, +3, +4, and +5). Cells were either scraped and processed for TCID_50_ assays or scraped with ice-cold RIPA buffer and processed for Western blotting. Uninfected cells and DMSO-treated cells infected for 6 h were used as controls. The ratios of the 2C/LB1 signals were quantified by using ImageJ, and data were plotted as a percentage compared to the values for the 6-h DMSO control.

### Immunofluorescence microscopy.

Cells were grown on coverslips and pretreated with DMSO or CRT0066101 at 5 μM for 1 h at 37°C. Human transferrin conjugated to Alexa Fluor 488 (Thermo Fisher Scientific) was added at a 75-μg/ml final concentration, and the cells were incubated at 37°C for 1 h in the presence of DMSO or CRT0066101. Cells were fixed with 4% formaldehyde (FA) and washed with PBS. After quenching of the residual FA with 0.1 M glycine, cells were washed with PBS, permeabilized with 0.1% Triton X-100 at RT, and washed with PBS again. After blocking in PBS–5% FBS, cells were incubated overnight with a mouse anti-GM130 antibody (BD Pharmingen) and for 45 min at RT with a donkey anti-mouse antibody coupled to Alexa Fluor 546 (Jackson ImmunoResearch). Cells were washed in PBS, and coverslips were mounted with ProLong Gold antifade reagent (Invitrogen). Coverslips were analyzed by using an LSM 5 Pascal laser scanning microscope (Carl Zeiss). Transferrin quantification is shown as the fluorescence density per cell. Cells from 5 different fields of either DMSO- or CRT0066101-treated cells from three independent experiments were quantified (∼250 cells per field). Total transferrin fluorescence was quantified by using ImageJ. The immunofluorescence staining protocol used for Fig. 2 and Fig. 5 is the same as described above but with the specific antibodies mentioned in the legends.

### Quantitative real-time PCR.

Cells were pretreated for 1 h at 37°C with CRT0066101 or the DMSO vehicle alone at the indicated concentrations. After adsorption of HRV16 or UV-inactivated HRV16 for 1 h at RT, cells were washed with PBS, fresh medium was added, and the cells were incubated for 20 h at 37°C. Cells were lysed with RLT buffer (Qiagen) supplemented with β-mercaptoethanol (Sigma) at a 1:200 dilution. mRNA extraction was performed by using the RNeasy minikit (Qiagen) according to the manufacturer's instructions. One microgram of mRNA was reverse transcribed for cDNA synthesis for 1 h at 37°C by using the Omniscript RT kit (Qiagen). Quantification of the levels of the different mRNAs of interest was conducted by using specific primer (Invitrogen) and probe (Eurofins) sequences, as follows: HRV forward primer 5′-GTGAAGAGCCSCRTGTGCT-3′ (50 nM), HRV reverse primer 5′-GCTSCAGGGTTAAGGTTAGCC-3′ (300 nM), HRV probe 5′-TGAGTCCTCCGGCCCCTGAATG-3′ (100 nM), OAS forward primer 5′-CTGACFCTGACCTGGTTGTCT-3′ (900 nM), OAS reverse primer 5′-CCCCGGCGATTTAACTGAT-3′ (900 nM), OAS probe 5′-CCTCAGTCCTCTCACCACTTTTCA-3′ (100 nM), 18S forward primer 5′-CGCCGCTAGAGGTGAAATTCT-3′ (300 nM), 18S reverse primer 5′-CATTCTTGGCAAATGCTTTCG-3′ (300 nM), and 18S probe 5′-ACCGGCGCAAGACGGACCAGA-3′ (100 nM). Analysis was performed by using QuantiTect Probe PCR master mix (Qiagen) and the LightCycler 480 real-time PCR system (Roche). For absolute quantification, the level of each gene was normalized to the level of 18S rRNA, and the exact number of copies of the gene of interest was calculated by using a standard curve generated by the amplification of plasmid DNA.

### PKD1 plasmid generation and transfection.

Human PKD1 cDNA was amplified by PCR from a gateway entry vector, pENTR223.1 (GE Healthcare), and cloned into a pRK5 plasmid with a Myc tag at the N terminus (Clontech) by standard molecular biology techniques. The following PKD1 primers (with the restriction sites indicated in parentheses) were used: 5′-TATATAGGATCCAGCGCCCCTCCGGTCCTG-3′ (BamHI) and 5′-TACGTAGAATTCCTAGAGGATGCTGACACG-3′ (EcoRI). All the mutations were made with a QuikChange II XL site-directed mutagenesis kit (Agilent Technologies) using the generated pRK5-Myc-PKD1 plasmid and the following primer pairs: 5′-TTGGAAATCGTAATTTGTCAATGATTGCAATAGCTACATCTCTTCCTGTTTTACG-3′ and 5′-CGTAAAACAGGAAGAGATGTAGCTATTGCAATCATTGACAAATTACGATTTCCAA-3′ for Myc-PKD1-K612A (KD), 5′-CTTGAGCCACCCTTGGTAGGAATTCCTGCAGA-3′ and 5′-TCTGCAGGAATTCCTACCAAGGGTGGCTCAAG-3′ for Myc-PKD1-ΔCT (deletion from amino acids [aa] 845 to 918), 5′-CCTCGGTGAGCGTGTCGCCATCCTCTAGGAATTC-3′ and 5′-GAATTCCTAGAGGATGGCGACACGCTCACCGAGG-3′ for Myc-PKD1-S916A, and 5′-CGAAGAGGAAAAGCAGCCTTATGCCCGTCATTCC-3′ and 5′-GGAATGACGGGCATAAGGCTGCTTTTCCTCTTCG-3′ for Myc-PKD1-ΔPH (deletion from aa 427 to 547). All the plasmid sequences were verified by DNA sequencing.

The transfection of HeLa cells with the generated PKD1 plasmids was performed by using FuGENE HD transfection reagent (Promega) at a ratio of 1.75:0.5 (microliters of FuGENE reagent/micrograms of DNA) and doubling the volume of the transfection mix per well compared to that specified by the manufacturer's instructions. The transfection efficiency was determined to be 70 to 80% and was controlled in each experiment by staining cells with an anti-Myc antibody and confocal microscopy. HeLa cells were transfected for 18 h and then infected with HRV16 at an MOI of 1 for 1 h, allowing replication to proceed for 6 h. The cells were then harvested, and cell lysates were prepared to determine endpoint titers by a TCID_50_ assay. Transfected but uninfected cells were harvested in parallel for Western blotting and immunofluorescence staining to analyze the expression, transfection efficiency, and phenotype of all the constructs.

### Statistical analysis.

Data are presented as means ± SEM. Statistical analysis was performed by using GraphPad Prism 6 software. For the TCID_50_ assay, Western blot quantification (Fig. 4B), and qRT-PCR data, the difference between groups was estimated by using one-way analysis of variance (ANOVA) with Dunnett's *post hoc* test. The difference in transferrin uptake between DMSO- and CRT0066101-treated cells (Fig. 4D) was determined by a two-tailed *t* test. For viral replication in PKD knockout cells (Fig. 6C), one-way ANOVA (95% confidence) was performed, showing significant differences among the means (*P* = 0.0131). A *post hoc* analysis was performed by using a two-tailed *t* test (95% confidence). The only statistically significant difference was between wt HAP1 and clone 001 (*P* = 0.0488). Differences between DMSO-treated cells and CRT0066101-treated cells at each time point in Fig. 7C were determined by two-way ANOVA with Sidak's *post hoc* test. In all the figures, asterisks are used to indicate statistically significant differences observed in comparisons of different experimental conditions.

## Supplementary Material

Supplemental material
