# Supplementary material for: Investigation of the Role of Protein Kinase D in Human Rhinovirus Replication
Source: J Virol. 2017 Apr 13;91(9):e00217-17. doi: 10.1128/JVI.00217-17 (PMC5391474; doi:10.1128/JVI.00217-17)
Supplement: Supplemental material [file supp_91_9_e00217-17__index.html]

Investigation of the Role of Protein Kinase D in Human Rhinovirus Replication — Supplemental material 

# Investigation of the Role of Protein Kinase D in Human Rhinovirus Replication

## Supplemental material

- Supplemental file 1 -

  Table S1 (Inhibition of various kinases by CRT0066501, CRT0066101, and XX-050.)

  PDF, 522K
