# Supplementary material for: Investigation of the Role of Protein Kinase D in Human Rhinovirus Replication
Source: J Virol. 2017 Apr 13;91(9):e00217-17. doi: 10.1128/JVI.00217-17 (PMC5391474; doi:10.1128/JVI.00217-17)
Supplement: Supplemental material [file JVI.00217-17_zjv999182546s1.pdf]

Supplementary Table S1

Inhibition of various kinases by CRT0066501, CRT0066101 and XX-050. Data shown as the % inhibition of each kinase by 1µM of CRT0066101, CRT0066051 and XX-050 in *in vitro* kinase assays. 0-50% inhibition (green), 50-90% inhibition (yellow), >90% inhibition (red).

	CRT0066101@1µM	CRT0066051@1µM	XX-50@1µM
CHKA	1	2	0
CHKB	3	0	-9
DGKB	-9	-8	-5
DGKG	-3	-18	-17
DGKZ	-1	3	0
PIK3CA	1	-12	-23
PIK3CB	-9	-12	10
PIK3CG	29	12	15
PIK3CA E524K + p85	31	13	4
PIK3CA E545K + p85	29	7	0
PIK3CA / p85 (h)	-1	-1	3
PIK3CB / p85 (h)	0	0	-4
PIK3CG / p85 (h)	-1	0	1
PI4K2A	4	0	0
PIK4B	33	12	12
PIK4A	-11	-14.3	-4.8
PIP4K2A	-22	-24	-29
SPHK1	19	17	24
SPHK2	20	21	8
Abl(h)	-13	38	10
Abl(T315I)(h)	5	45	22
ALK(h)	3	16	35
AMPKα1(h)	17	5	55
ARK5(h)	49	-20	98
Aurora-A(h)	-1	-10	4
Axl(h)	39	31	85
Blk(m)	12	3	3
Bmx(h)	26	74	45
BRK(h)	17	95	67
CaMKI(h)	2	21	56
CaMKIV(h)	26	6	60
CDK1/cyclinB(h)	63	14	59
CDK2/cyclinA(h)	53	-12	41

CDK5/p35(h)	95	9	94
CHK1(h)	-2	6	36
CHK2(h)	26	43	71
CK1γ1(h)	-5	-6	49
CK1γ2(h)	0	-9	81
CK1γ3(h)	-3	-14	61
CK1δ(h)	13	12	89
CK2(h)	19	-1	14
cKit(D816H)(h)	51	29	82
cKit(V560G)(h)	24	66	93
CSK(h)	7	27	-11
c-RAF(h)	4	-2	-4
cSRC(h)	3	-11	32
DAPK1(h)	-8	-14	23
DDR2(h)	-3	-10	-3
DYRK2(h)	94	0	11
EGFR(h)	-6	1	4
EGFR(L858R)(h)	-1	0	5
EGFR(L861Q)(h)	10	39	20
EGFR(T790M)(h)	-2	76	24
EGFR(T790M,L858R)(h)	-3	47	31
EphA2(h)	2	3	34
EphA7(h)	-9	-8	34
EphB4(h)	25	12	36
ErbB4(h)	3	18	12
FAK(h)	-11	3	42
Fer(h)	-25	2	10
Fes(h)	-20	-22	45
FGFR1(h)	-30	31	-5
FGFR2(h)	5	12	16
FGFR3(h)	-2	11	11
FGFR4(h)	-2	-10	-8
Flt1(h)	-6	54	64
Flt3(D835Y)(h)	80	15	100
Flt3(h)	4	3	99
Flt4(h)	22	35	94
Fms(h)	12	20	72
GSK3β(h)	18	7	36
Hck(h)	4	44	66
HIPK1(h)	23	-16	52
HIPK2(h)	55	4	79
HIPK3(h)	14	-14	50
IGF-1R(h)	-14	-2	13
IKKα(h)	66	5	92
IR(h)	-22	8	27
JAK2(h)	10	11	13
KDR(h)	13	15	70
LIMK1(h)	0	2	16
LKB1(h)	-13	-6	9
MAPK2(h)	-9	-12	-15

MAPKAP-K2(h)	-9	-9	-1
MEK1(h)	9	23	59
MELK(h)	55	23	82
Mer(h)	66	29	101
Met(h)	12	24	45
MST2(h)	20	12	66
MST3(h)	-6	8	-14
NEK2(h)	0	-8	3
p70S6K(h)	31	20	68
PAK2(h)	9	13	17
PAK4(h)	4	-7	71
PDGFRα(h)	14	15	15
PDGFRα(D842V)(h)	61	11	74
PDGFRβ(h)	-1	-11	13
PDK1(h)	-17	-31	27
Pim-1(h)	98	2	94
PKBα(h)	-6	-11	18
PKCα(h)	4	-1	-3
PKCβI(h)	11	0	-8
PKCβII(h)	-1	0	-1
PKCγ(h)	7	-5	-13
PKCδ(h)	28	0	11
PKCε(h)	12	-7	-7
PKCη(h)	4	-7	-5
PKCι(h)	10	3	6
**PKCμ(h)**	98	99	92
PKCθ(h)	6	-1	15
PKCζ(h)	-8	-19	-12
**PKD2(h)**	96	99	83
PRK2(h)	77	-17	65
Ret(h)	0	-3	59
ROCK-I(h)	4	-5	10
ROCK-II(h)	28	-2	27
Ron(h)	-8	-4	7
Ros(h)	-13	-10	-20
Rsk1(h)	48	-21	47
Snk(h)	-1	-6	-8
TAK1(h)	-6	0	33
Tie2 (h)	4	44	12
TrkA(h)	23	4	90
TrkB(h)	15	-18	89
Yes(h)	40	22	83
ZAP-70(h)	-19	-3	-9
ZIPK(h)	7	13	53
